# Murder

**Published:** 1869-06

**Authors:** 


Murder.—A slip giving the report of deaths in the city of Providence, R. I., during the month of May, 1869, has been sent us with the following passages marked:

"There were two murders in the month of May, which are only counted in the above table as one death, under the head of *criminal abortion.* The victims were a young, healthy, married woman and her unborn child. From some foolish reason, at any rate with no good and sufficient reason, she consented to do violence to the laws of her being, to prevent the high object of the marriage relation, and to become accessory to the murder of her own offspring. In so doing she lost her own life. It would be well if her death might be a warning to others, and if all would remember that this crime can never be done with safety. It is always murder of one human being; there is always possible danger to the life of the mother; there is always certain injury to her health and happiness.

"The murderer in this case received twenty-five dollars for his services in killing two human beings. He assured the deluded woman that there was no danger, but exacted from her a solemn promise that 'whatever might happen, she would not tell any one of his crime.' Edwin M. Snow, M. D.,

Superintendent of Health and City Registrar."

—*Boston Medical Journal.*

